# Building on Lessons Learned in a Mobile Intervention to Reduce Pain and Improve Health (MORPH): Protocol for the MORPH-II Trial

**DOI:** 10.2196/29013

**Published:** 2021-07-19

**Authors:** Jason Fanning, Amber K Brooks, Katherine L Hsieh, Kyle Kershner, Joy Furlipa, Barbara J Nicklas, W Jack Rejeski

**Affiliations:** 1 Department of Health and Exercise Science Wake Forest University Winston-Salem, NC United States; 2 Wake Forest School of Medicine Winston-Salem, NC United States

**Keywords:** aging, physical activity, sedentary behavior, weight loss, chronic pain, mHealth

## Abstract

**Background:**

Engaging in sufficient levels of physical activity, guarding against sustained sitting, and maintaining a healthy body weight represent important lifestyle strategies for managing older adults’ chronic pain. Our first Mobile Health Intervention to Reduce Pain and Improve Health (MORPH) randomized pilot study demonstrated that a partially remote group-mediated diet and daylong activity intervention (ie, a focus on moving often throughout the day) can lead to improved physical function, weight loss, less pain intensity, and fewer minutes of sedentary time. We also identified unique delivery challenges that limited the program’s scalability and potential efficacy.

**Objective:**

The purpose of the MORPH-II randomized pilot study is to refine the MORPH intervention package based on feedback from MORPH and evaluate the feasibility, acceptability, and preliminary efficacy of this revised package prior to conducting a larger clinical trial.

**Methods:**

The MORPH-II study is an iteration on MORPH designed to pilot a refined framework, enhance scalability through fully remote delivery, and increase uptake of the daylong movement protocol through revised education content and additional personalized remote coaching. Older, obese, and low-active adults with chronic multisite pain (n=30) will be randomly assigned to receive a 12-week remote group-mediated physical activity and dietary weight loss intervention followed by a 12-week maintenance period or a control condition. Those in the intervention condition will partake in weekly social cognitive theory–based group meetings via teleconference software plus one-on-one support calls on a tapered schedule. They will also engage with a tablet application paired with a wearable activity monitor and smart scale designed to provide ongoing social and behavioral support throughout the week. Those in the control group will receive only the self-monitoring tools.

**Results:**

Recruitment is ongoing as of January 2021.

**Conclusions:**

Findings from MORPH-II will help guide other researchers working to intervene on sedentary behavior through frequent movement in older adults with chronic pain.

**Trial Registration:**

ClinicalTrials.gov NCT04655001; https://clinicaltrials.gov/ct2/show/NCT04655001

**International Registered Report Identifier (IRRID):**

PRR1-10.2196/29013

## Introduction

Physical activity is a key behavioral medicine for supporting quality of life [1] and managing chronic pain [2]. A healthy activity profile for all individuals, including those with chronic pain, involves achieving a sufficient volume of physical activity, which can reduce pain sensitivity and pain intensity [3], while minimizing the presence of prolonged bouts of sitting, which can exaggerate pain symptoms [4,5]. Promoting these behaviors is especially challenging among older adults with chronic pain whose pain symptoms act as a potent daily barrier to movement [6,7]. In fact, Chastin and colleagues [8] reported that pain was the most potent driver of sedentary behavior in a sample of community-dwelling older adults. Often exercise and sedentary behaviors are treated independently: Participation in a single daily bout of sustained and intense exercise leaves much of the day for prolonged sitting [9] and may even lead to compensatory increases in sitting [10]. This is particularly problematic from the perspective of weight management in older adults with chronic pain. Obesity is powerfully associated with pain [11], and physical activity is considered a central component to weight management [12]. However, the tendency for older adults to engage in compensatory sitting following structured exercise can result in a counterproductive reduction in daily energy expenditure in the short term, which may in turn contribute to long-term weight gain [13-16].

Modifying both exercise and sedentary behavior requires unique behavior change strategies. Motivating exercise involves supporting the individual to engage and sustain a single challenging behavior each day in the face of barriers (eg, changing life events, fatigue, injury) [17,18]. Breaking up prolonged sitting requires an individual to develop an awareness of a habitual behavior (ie, one that occurs normally with little thought) [19] and maintain the motivation needed to self-regulate this behavior consistently across the day [20,21]. It is notable that many have difficulty recognizing how much they sit—as indicated by our tendency to dramatically underreport daily sitting time [22,23]—and so objective self-monitoring tools are especially important for altering sedentary behaviors [24].

A novel approach to addressing physical activity and sedentary time in tandem is to focus on the accumulation of physical activity with an emphasis on distributing this activity throughout the day, thereby indirectly breaking up sustained sitting bouts [20]. A distributed movement goal aligns with the second edition of the United States Federal Physical Activity Guidelines [1], which emphasize a “move more, more often” approach to physical activity and remove the need to separately target structured exercise and sitting behaviors. We illustrated this approach in the Mobile Intervention to Reduce Pain and Improve Health (MORPH) pilot trial wherein older adults with chronic pain attempted to lose weight and improve function through caloric restriction and increased physical activity via the accumulation of activity throughout the day [20]. Compared with a control condition, 12 weeks of MORPH resulted in weight loss, improved function, and reduced sedentary time and pain intensity [25]. We also identified key limitations in the technological tools and coaching model used in MORPH (detailed in the Design Consideration section). Thus, the purpose of MORPH-II is to refine the MORPH intervention toolset and coaching model based on feedback from the first implementation of MORPH prior to testing the package in a large and costly clinical trial [26]. The primary aim of this study is to determine the feasibility and acceptability of this refined MORPH intervention delivered entirely remotely. We will also explore the effect of MORPH-II for increasing time spent active while reducing sedentary time relative to a no-contact control.

## Methods

### Study Overview

A description of the full 24-week study period is detailed below. Protocols were reviewed and approved by a university institutional review board in January 2021. All eligible and interested individuals will complete an approved informed consent prior to participation.

### Participants

MORPH-II is a randomized controlled pilot study in which participants (minimum n=30, allowing for at least 3 waves with refinements between) will enter the study in waves of 8 to 12 individuals (4 to 6 per condition). Eligible participants must use a study-provided iPad (Apple Corp) tablet, own a smartphone to facilitate preintervention in-home testing, have pain on most days during the previous 3 months, have no contraindication for participation in exercise, and be aged 55 to 85 years, obese (BMI: 30 to 45 kg/m^2^ based on self-reported height and weight and corrected via the Shields equation) [27], weight-stable (ie, no weight loss or gain of more than 5% of body weight in the past 6 months), and low-active (ie, engaging in less than 2 days per week of structured physical activity for at least 20 minutes). Exclusion criteria will be inability to walk without assistive devices for short distances or cognitive impairment as indicated by a modified telephone interview for cognitive status [28] score below 31. Individuals will be recruited to participate in a 2-group randomized controlled trial wherein they will be assigned to either the 24-week mobile health (mHealth) and telecoaching intervention (12 weeks of active intervention, 12 weeks of follow-up) or a control group. The primary outcomes for this pilot study include feasibility and acceptability, and secondary outcomes include daily steps and sedentary time as assessed by the ActivPAL 4 (PAL Technologies Ltd).

### Recruitment, Screening, and Randomization

We will recruit participants using several methods. We will leverage targeted mass mailings via postcards to individuals across North Carolina fitting demographic characteristics of our target population. We will place phone calls or send emails to individuals identified in regional research databases comprising individuals who inquired about other research studies and opted to be contacted for future research opportunities. We will advertise the study using digital and print newspapers within the state of North Carolina and regional newsletters that advertise research opportunities and medical news for those who opt in. All individuals who respond to our recruitment strategies will be screened via telephone for eligibility. Those who pass phone screening will be scheduled for a phone visit and mailed a packet containing an informed consent document and questionnaire forms, and the informed consent will be completed via telephone in accordance with our review board policy before any data collection occurs. The completed baseline questionnaires will be returned in postage-paid envelopes. After completing paper questionnaires and the phone-based cognitive assessment [28], participants will complete a remote Short Physical Performance Battery (SPPB) [29]. Eligible participants will then be randomized to the MORPH-II intervention or a control condition using a web-based randomization scheme.

### Intervention Design Modifications

**Figure 1:**
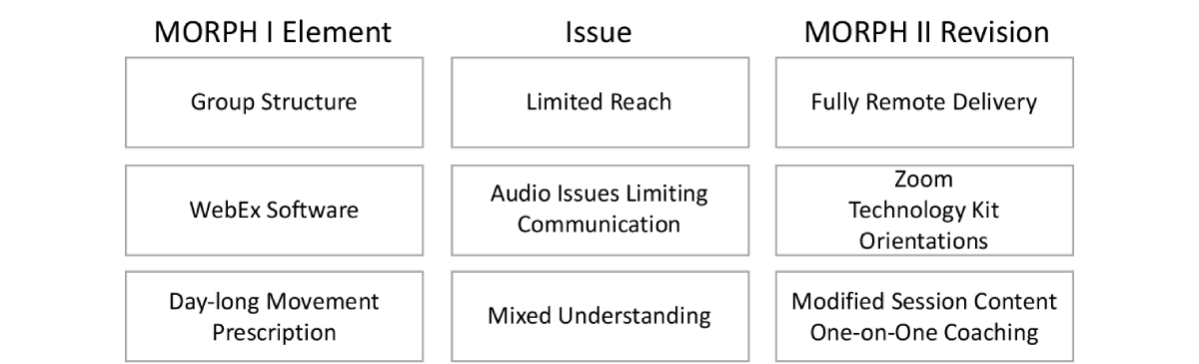
Design modifications for MORPH-II. MORPH: Mobile Intervention to Reduce Pain and Improve Health.

The first iteration of MORPH revealed key design issues we believe hampered uptake of the movement prescription and limited its potential for widespread delivery. These are noted in the following sections in detail and depicted in Figure 1.

### Study Orientation and Tool Set

**Figure 2:**
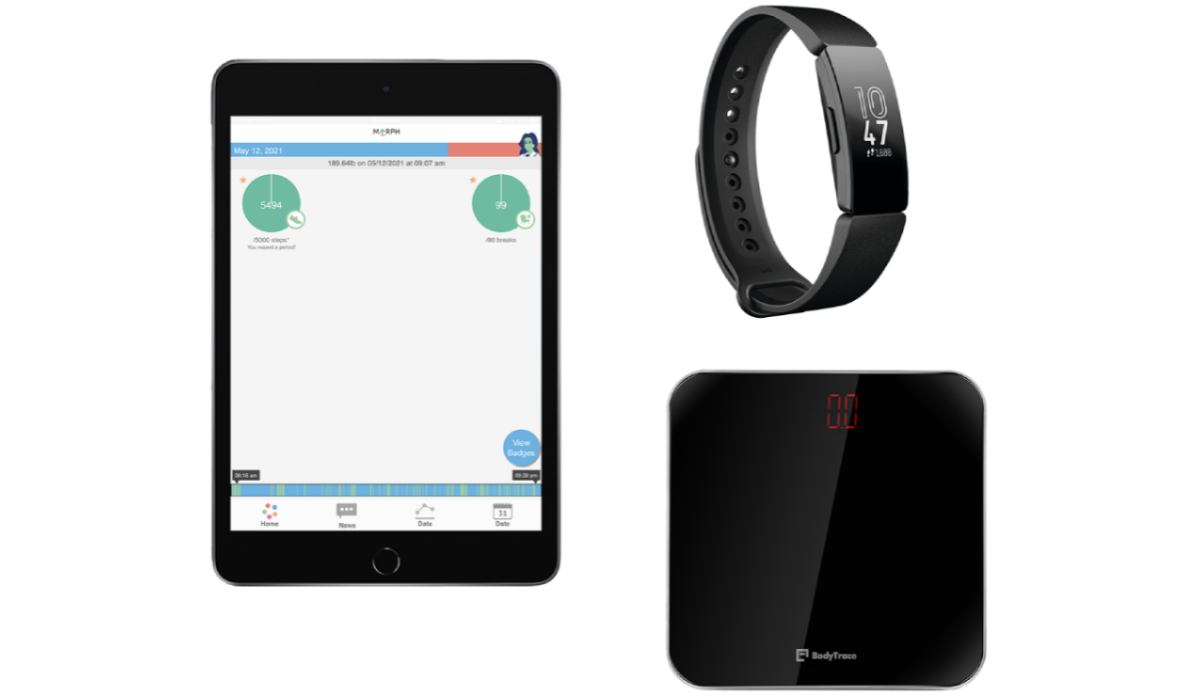
Intervention condition technology tools include iPad equipped with the MORPH companion app, Fitbit app, and teleconference software; BodyTrace cellular scale; and Fitbit Inspire activity monitor. Control participants receive the Fitbit monitor only.

MORPH-II comprises a focused 12-week program followed by a 12-week no-contact maintenance period wherein intervention participants will be encouraged to sustain their weekly group contacts without researcher support. The first iteration of MORPH was limited in its ability to scale, as all testing was conducted in person, as were the first 3 group meetings. MORPH-II addresses these limitations by conducting the program remotely and employing remote testing and orientation appointments. This orientation appointment will occur over the telephone and video conference software and will allow the behavioral interventionist to introduce the study technology tools (Figure 2) and provide initial information on the study’s approach to accumulating physical activity and dietary weight loss for those in the active arm. This will occur after the baseline physical activity data collection to avoid any contamination from the Fitbit activity monitor. Those randomized to the control condition will receive the scale and Fitbit activity monitor as well.

### MORPH-II Intervention

**Figure 3:**
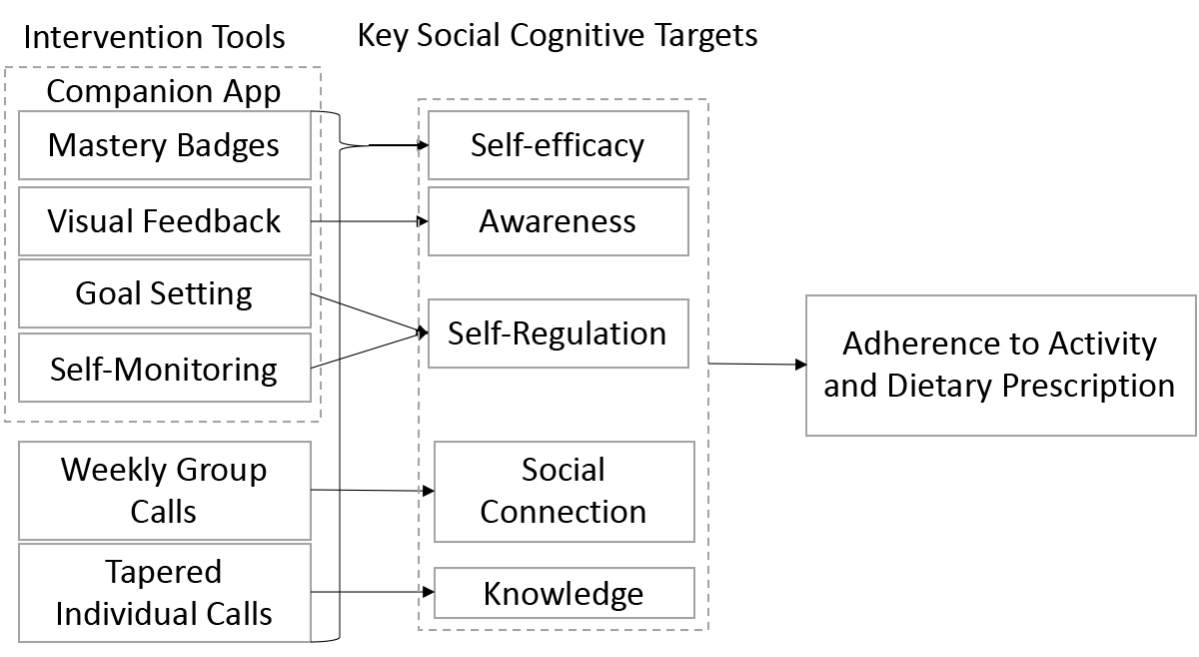
MORPH-II intervention model is based on social cognitive and self-determination theories.

**Figure 4:**
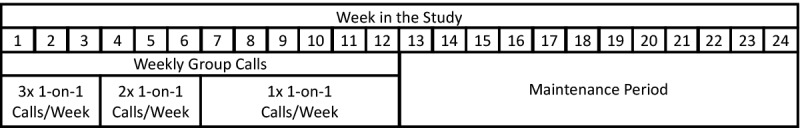
Study timeline.

The model underlying the MORPH-II intervention package is depicted in Figure 3. MORPH-II is informed by our previous work in theory-based mHealth activity promotion in older adults [20,25,30,31], grounded in social cognitive [32] and self-determination [33] theories. The intervention involves an emphasis on regular group meetings as a motivational tool and for developing knowledge related to the importance of frequent physical activity and weight loss. The group meetings are supported by brief one-on-one meetings to reinforce the distributed movement goals and provide technology support. The MORPH-II intervention also involves the use of an mHealth toolset to instill an awareness of habitual sedentary behaviors, allow for unique goal setting and self-monitoring, and foster the development of self-efficacy via mastery badges [30], which act to cue successful experiences. The MORPH-II study timeline is depicted in Figure 4.

### Group-Mediated Teleconference Contacts

During the active intervention, participants will meet via Zoom teleconference software once weekly as a group. In a key design issue in the first iteration of MORPH, the Webex (Cisco) software often muted speakers when there was background noise, an important limitation in a group-mediated program. We have selected Zoom and will employ headphones with built-in microphones to foster more natural communication in group sessions. These sessions help to form social bonds to support long-term behavior change, foster self-efficacy through modeling, allow participants to share successes, and provide a venue for troubleshooting barriers as they arise. Within these group sessions, the behavioral interventionist will also work with participants to develop the basic skills needed for successful daily movement and weight loss (eg, regular self-monitoring, food tracking, use of the app each day). A more thorough description of the group-mediated model used in this study and a list of session topics is provided in Multimedia Appendix 1.

### Movement and Weight Loss Goals

The movement component of MORPH involves increasing time spent in light and moderate-to-vigorous physical activity across the day through a focus on accumulating all forms of stepping activity while reducing the presence of sustained sitting bouts. This is supported in the study mHealth app via unique daily step goals that must be accumulated across 3 periods of the day, visualization of movement patterns, and goals related to the number of shifts (ie, breaks) between nonmoving and moving periods. The distal goals of the activity component are increasing daily steps in the range of 5000 to 10,000 steps based on individual abilities and distributing these steps throughout the day.

The content for the dietary weight loss intervention is based on our extensive experience in community-based trials [20,25,31,34]. The aim of the dietary intervention is to drive weight loss through caloric restriction while maintaining a nutritious and balanced diet. Individual goals for caloric intake will be prescribed to achieve an energy deficit of ~400 kcal/d from daily weight maintenance energy requirements (resting energy expenditure × activity factor of 1.3 for sedentary adults). During an initial orientation appointment, participants will be introduced to key concepts in nutrition (eg, self-monitoring, importance of protein consumption). Participants will work with the intervention team to set weekly calorie goals based on food logs collected during the week, behavioral challenges that arose during the prior week, and weight loss progress. The lowest caloric goal prescribed will be 1100 kcal/d for women and 1200 kcal/d for men. In the first MORPH study, the nutrition content was front-loaded, causing many participants to have difficulty conceptualizing the daily movement goals. Thus, we have reduced the focus on dietary topics such that nutrition content is introduced during an orientation and then covered in depth during weeks 7 to 12. Nutrition topics include eating to maintain satiety, managing a calorie balance on a weekly basis (eg, to account for celebratory eating events), and maintaining a healthy diet in the long term. This approach produced a significant weight reduction of nearly 3 kg compared with the control condition in the 12-week MORPH study [25]. Sessions also include nutritional education, teaching and reinforcement of self-regulatory skills, exposure to mindfulness-based stress reduction and pain management, and strategies that optimize social connection.

### MORPH Companion App and Individual Coaching Calls

Participants in the intervention condition will be urged to engage with the MORPH companion app at least 3 times daily (morning, midday, and evening). This progressive web app will come preinstalled on the participant’s iPad and will stream data in near real time from the Fitbit activity monitor and BodyTrace scale. The primary functions of the app are to provide immediate feedback on behavioral successes (thus supporting self-efficacy) via highly specific mastery badges, allow for ongoing social connection between weekly meetings via an in-app chat function, foster self-monitoring of weight via a connected smart scale, and instill an awareness of one’s patterns of movement and sitting behaviors. This is primarily achieved through a timeline bar (Figure 2, lower image) wherein active minutes collected from the Fitbit are displayed in green and inactive minutes are displayed in blue. Participants aim for a “tree rings” profile on their timeline bar such that stripes of green movement are evenly dispersed throughout the day, interrupting any lengthy blue sitting bouts. Additionally, participants receive periodic step goals, which are modified step goals designed to disincentivize a single bout of exercise surrounded by sitting time. Here, participants are able to achieve up to 40% of their daily goal before 12:30 PM, again between 12:30 PM and 5:30 PM, and once more after 5:30 PM, necessitating at least 20% of their daily steps during any one period.

To reinforce these unique activity goals, MORPH-II participants will receive brief one-on-one coaching calls in a tapered fashion, as noted in Figure 1. These are designed based on feedback from the first iteration of MORPH and are intended to foster a stronger understanding of the daylong movement prescription—something that appeared unintuitive to some participants in our first iteration of MORPH. During these conversations, the coach and participant will view the participant’s timeline bar from the previous day. They will discuss whether the participant felt they were successful in their daylong movement goal and troubleshoot periods of sustained sitting visible on the bar. The pair will work to develop actionable goals designed to enhance daylong movement over subsequent days, and these goals will be reviewed in the next one-on-one call. To facilitate rapid uptake of these goals, we have front-loaded these calls such that there will be 3 of these calls each week during weeks 1 to 3, 2 during weeks 4 to 6, and 1 during weeks 6 to 12 (Figure 4).

### Maintenance Recommendations

One key advantage of fully remote telehealth delivery of group meetings is that participants may sustain their sessions without the need for a physical meeting space. During the final weeks of the focused intervention period, participants will receive instruction on continuing their weekly group calls without the assistance of the intervention team, emphasizing the importance of social connection for behavioral maintenance. No contact will be made with participants except for the purpose of scheduling and assessment during weeks 12 to 24. We will continue to collect app use and Fitbit data during this period.

### Control Condition

Those in the control condition will be offered a print-based version of the intervention upon completion of the final testing time point. This will include a pamphlet detailing activity monitoring and how to use the Fitbit to monitor movement across the day. The pamphlet will also include a guide to food tracking and safe caloric restriction goals. These individuals will also receive the BodyTrace cellular scale and instruction on the value of daily weighing for weight management.

### Measures

Baseline demographic data, medical information, and medication use will be recorded based on participant self-report. For the purposes of screening, BMI will be estimated using self-reported height and weight. The Shields adjustment [27] will be applied using the following formulas:

*BMI_Males_* = –0.29227 + *X_Self-reported BMI_* × 1.03239

*BMI_Females_* = 0.10927 + *X_Self-reported BMI_* × 1.02584

Adverse events will be assessed by questioning participants at each assessment visit and during weekly telecoaching calls.

To assess physical function, we will conduct the SPPB [29] via video conference and recorded video prior to the start of the intervention, after week 12, and after week 24. As described elsewhere, participants will receive a testing kit via mail including a camera, rope and tape to mark a walking course, and instructions. The participant will meet a blinded tester via video conference. The tester will review the testing setup with the participant, and then prior to each task will provide verbal and written instructions and ask whether the participant feels safe completing the task. We will also require a second individual such as a family member or friend to be present and in the same room during any SPPB appointment to assist in case of a fall. We will provide this safety partner with an information sheet listing their responsibilities—to be in the same room and actively observing the participant during the appointment—as well as the requirement to have a phone on hand in order to call 911 if necessary. The kits will be returned by mail, and the camera footage will be used for test scoring. We will also gather self-reported disability using the Pepper Assessment Tool for Disability [35].

We will assess pain symptomology using the Patient-Reported Outcomes Measurement Information System [36] pain intensity (3 item) and pain interference (8 item) scales. We will monitor body weight throughout the study in the home using the BodyTrace cellular-enabled scale. We will use the ActivPAL 4 monitor to assess treatment effects on daily sedentary behaviors (time spent sedentary, sedentary breaks, average bout length) and physical activity (steps, minutes of total activity, minutes of moderate-to-vigorous activity). Participants will be asked to wear the devices continuously for 7 consecutive days at each time point. Data will be downloaded at the end of each 7-day period and cleaned and summarized for statistical analyses. We will also obtain daily Fitbit data throughout the study, which will provide ongoing assessments of physical activity and sitting behavior.

We will assess self-efficacy expectations related to physical activity [37,38] and outcome expectations for physical function and appearance [39]. Perceived stress will be evaluated with a short-form of the perceived stress scale [40], and control in resisting food using the power of food scale [41]. Depression will be assessed using the Center for Epidemiological Studies Depression scale [42], and health-related quality of life using the 36-item Short Form quality of life scale [43]. Next, we will capture the extent to which participants’ self-determinative needs are being met using the Needs Satisfaction Scale specific to this study [44]. We will assess an individual’s life space using a modified life space questionnaire [45]. The Pittsburgh Sleep Quality Index [46] will be used to assess sleep at each assessment visit. Fatigue will be measured using the Functional Assessment of Chronic Illness Therapy fatigue scale [47]. Cognitive function will be assessed during screening using the modified telephone interview for cognitive status [48]. Those scoring below 32 will be exclude from the study. We will use the Hopkins verbal learning test to assess word recognition and delayed recall as it has been validated for use over the phone.

Recognizing the dynamic relationship between pain and health behaviors, we will collect ecological momentary assessments [49] of pain, sleep quality, and affect throughout the program. To minimize participant burden, we selected well-used single-item assessments of each of these domains. Sleep quality will be assessed once daily via items from the Pittsburgh Sleep Quality Index [46] that we have used previously to investigate the relationship between sleep quality and activity behavior [50]. Specifically, participants will respond to “My sleep quality last night was...” on a 5-point scale ranging from very poor to very good. They also self-report the number of hours they slept the previous night. Affect, pain, and pain medication use will be assessed 6 times daily (1 unprompted waking survey and 5 prompted surveys delivered between late morning and midevening). Affect will be assessed using the single-item Feelings Scale that was developed and validated by Hardy and Rejeski [51]. This is a widely used assessment of affect, including in ecological momentary assessment studies [52]. Responses are provided on an 11-point scale ranging from feeling very bad to very good. Participants will be asked to rate their level of pain right now on an 11-point numeric rating scale [53] with responses ranging from none to severe. Finally, participants note whether they have taken a medication to manage their pain since the previous survey. We will collect these for 1 week at the beginning of each month of the intensive intervention phase (ie, weeks 1, 5, and 9), with surveys prompted via push notification and completed within a study web application.

Following weeks 12 and 24, those in the intervention condition will be asked to reply to several Likert-type and short-response items pertaining to their perceived use and usability of the app and acceptability of the intervention. Additionally, we will conduct a telephone guided interview with participants who received the active intervention after week 12 to collect qualitative data pertaining to study usability and acceptability. Finally we will collect the system usability scale [54] from intervention participants after week 12.

### Statistical Analyses

The primary aim of this study is to describe the feasibility (ie, recruitment yield, percentage attendance at one-on-one and group coaching sessions) and acceptability (ie, participant feedback, system usability) of the MORPH-II intervention. We will present descriptive statistics for each of these outcomes. We will also look for group differences in physical activity and sedentary time to explore whether effects are in the appropriate direction. We will apply the following generalized linear modeling approach:

*Y_FU_* = *β_0_* + *β_1_Y_BL_* + *β_2_Int* + covariates + *ε*

where *Y* represents the focal outcomes (sedentary time, time spent in physical activity); subscripts *FU* and *BL* denote follow-up and baseline measurements, respectively, at week 12; *Int* represents intervention status; *β* represents regression coefficient; and *ε* represents random error. We also plan to conduct similar analyses of additional outcomes of the randomized controlled trial (eg, weight loss and function and disability scores) using the aforementioned model. Finally, we will replicate these analyses on scores at week 24 to investigate the extent to which those in the intervention condition maintain their behavior. We will also present descriptive statistics related to app use over the full 24-week study period.

## Results

The study has received institutional review board approval and recruitment is ongoing as of January 2021. The study was registered at ClinicalTrials.gov [NCT04655001].

## Discussion

### Summary

Accumulating sufficient levels of physical activity and minimizing long bouts of sitting are widely recognized as important behavioral treatments for many health conditions, including chronic pain [3-5]. The development of effective and scalable interventions for promoting movement throughout the day is in its infancy, and as such it is vital that a careful iterative approach is used to identify effective intervention components while rectifying those that are ineffective. MORPH-II builds on an initial intervention framework [20,25] that demonstrated early success while bearing several important limitations. MORPH-II accomplishes the important step of further refining our initial intervention approach prior to testing it in a large and costly clinical trial. Our findings will serve as helpful guidance for other researchers working to intervene on sedentary behavior through frequent movement.

### Limitations

While we believe the remotely delivered MORPH-II study will represent an important step toward socially rich and scalable activity programming, we also recognize several limitations that will be addressed in future research. This pilot study is intended to refine a complex remote intervention framework and as such will not be powered as an efficacy study. Additionally, the program sessions are designed to be led by a trained behavioral coach. Therefore, future work would benefit by recruiting a sufficiently large sample to determine efficacy and by working with community partners to deliver the program more broadly.

## Appendix

Multimedia Appendix 1 Information on the group-mediated behavior change model used in MORPH-II.

## Abbreviations

mHealth: mobile health
MORPH: Mobile Intervention to Reduce Pain and Improve Health
SPPB: Short Physical Performance Battery
