# Supplementary material for: Building on Lessons Learned in a Mobile Intervention to Reduce Pain and Improve Health (MORPH): Protocol for the MORPH-II Trial
Source: JMIR Res Protoc. 2021 Jul 19;10(7):e29013. doi: 10.2196/29013 (PMC8329761; doi:10.2196/29013)
Supplement: Multimedia Appendix 1 [file resprot_v10i7e29013_app1.docx]

# Supplement

## The MORPH-II Group Mediated Sessions

The remote group-mediated sessions allow us to address key social cognitive mediators of behavior change, including (1) bolstering self-efficacy through modeling, highlighting successes, positive verbal persuasion, and education; (2) addressing negative outcome expectancies and (3) highlighting facilitators while addressing barriers through education and peer discussion. Additionally, these sessions provide a venue for education on effective self-monitoring and for heightening participants’ value they place on mobility and social connections. Finally, these sessions allow us to provide education and resources to prevent relapse and encourage behavioral maintenance. These sessions are led by intervention staff with experience conducting group-mediated sessions. Additionally, all intervention staff are trained to provide effective technology support and we aim to include at least two staff in each group call so that one may address technical challenges as they arise. Each session includes:

1. Housekeeping—Attendance and general goal review as participants filter in.
2. Breakout “Catch-Up” – Because videoconference calls make natural group conversation more difficult, we will start with 2–3-person breakout rooms to allow for social cohesion to occur. These will mostly center on discussion of challenges and success from previous weeks, plus brief conversation prompts (e.g., discuss one hobby and then present your partner’s hobby to the group).
3. Presentation of topic & discussion —After hearing from the breakout rooms, the facilitator will transition to the day’s topic. Participants will be encouraged to read their workbook content related to each week’s topic prior to attending the session so that the presentations can be brief, highlighting only the most relevant points for conversation. Participants will be asked to participate in several ways such as helping the group define and relate to the topic; giving examples related to the topic; problem solving; and actively listening and responding to ideas and thoughts from the other group members. After this period, participants will transition to exercise for the day.
4. Summary, Takeaways, and Reflection – Sessions will conclude with a group discussion of the weeks’ topic and a Mindfulness Reflections exercise, which are modeled on Mindfulness Based Relapse Prevention.

**Session topics:**

1. Freedom in Movement: Participants learn to define physical activity and receive an overview of the value of physical activity for health and well-being (especially in pain).

2. Move Across the Day…What Does That Mean? Participants take a deep dive on the rationale for a distributed movement prescription, especially with pain.

3. Stepping Up to the Challenge: Participants engage in an exploration of why physical activity can be a challenge, and how states like stress can interfere with physical activity.

4. Feelings, Thought and Behaviors, Oh My! Participants learn how thoughts and emotions affect behaviors and work to develop a deeper awareness of their own emotions.

5.The Curse and Power of Our Thoughts: Participants receive a more thorough overview of how fleeting thoughts and emotions affect behavior.

6. Relaxing to Heal Your Body: Participants receive a deep dive on the influence of stress on activity behavior and strategies for managing stress.

7. Portion Conscious and Taming Appetite: Participants discover how to gauge portions and sustain satiety.

8. Health Giants: Whole Grains, Fruits and Vegetables: Participants receive an overview of these heavy hitters in nutrition.

9. Mindful Meditation: After several weeks of practical mindfulness activities, this session focuses on the value of brief mindful meditation for sustaining behavior change.

10. Motivation and Goals: Participants work to outline their personal motivations and to handle periods of low motivation for movement or healthy eating.

11. Balancing a Balanced Diet for Weight Control: Participants learn how to value food quality, build a balanced meal, and mirror those who successfully keep weight off.

12. Final Thoughts: Participants debrief on their time in the program and are provided with tools to sustain their groups as they transition out of the structured period of MORPH.
